# Glucocorticoids Distinctively Modulate the CFTR Channel with Possible Implications in Lung Development and Transition into Extrauterine Life

**DOI:** 10.1371/journal.pone.0124833

**Published:** 2015-04-24

**Authors:** Mandy Laube, Miriam Bossmann, Ulrich H. Thome

**Affiliations:** Center for Pediatric Research Leipzig, Hospital for Children & Adolescents, Division of Neonatology, University of Leipzig, Leipzig, Germany; University Medical Center Utrecht, NETHERLANDS

## Abstract

During fetal development, the lung is filled with fluid that is secreted by an active Cl- transport promoting lung growth. The basolateral Na^+^,K^+^,2Cl^-^ cotransporter (NKCC1) participates in Cl^-^ secretion. The apical Cl^-^ channels responsible for secretion are unknown but studies suggest an involvement of the cystic fibrosis transmembrane conductance regulator (CFTR). CFTR is developmentally regulated with a high expression in early fetal development and a decline in late gestation. Perinatal lung transition is triggered by hormones that stimulate alveolar Na^+^ channels resulting in fluid absorption. Little is known on how hormones affect pulmonary Cl^-^ channels. Since the rise of fetal cortisol levels correlates with the decrease in fetal CFTR expression, a causal relation may be assumed. The aim of this study was to analyze the influence of glucocorticoids on pulmonary Cl^-^ channels. Alveolar cells from fetal and adult rats, A549 cells, bronchial Calu-3 and 16HBE14o- cells, and primary rat airway cells were studied with real-time quantitative PCR and Ussing chambers. In fetal and adult alveolar cells, glucocorticoids strongly reduced Cftr expression and channel activity, which was prevented by mifepristone. In bronchial and primary airway cells CFTR mRNA expression was also reduced, whereas channel activity was increased which was prevented by LY-294002 in Calu-3 cells. Therefore, glucocorticoids strongly reduce CFTR expression while their effect on CFTR activity depends on the physiological function of the cells. Another apical Cl^-^ channel, anoctamin 1 showed a glucocorticoid-induced reduction of mRNA expression in alveolar cells and an increase in bronchial cells. Furthermore, voltage-gated chloride channel 5 and anoctamine 6 mRNA expression were increased in alveolar cells. NKCC1 expression was reduced by glucocorticoids in alveolar and bronchial cells alike. The results demonstrate that glucocorticoids differentially modulate pulmonary Cl^-^ channels and are likely causing the decline of CFTR during late gestation in preparation for perinatal lung transition.

## Introduction

During fetal development the lung is filled with fluid which is actively secreted by lung epithelial cells [1]. This lung fluid has a discrete composition consisting of a high Cl^-^ and low protein concentration and is the product of an active Cl^-^ secretion into the lumen. Vectorial Cl^-^ transport drives water secretion into the lung which is crucial for lung expansion and thereby for growth of the developing airways and alveoli [1]. Alterations of fluid dynamics result in developmental lung anomalies. Tracheal obstruction and accumulation of lung fluid lead to lung hyperplasia whereas drainage of lung liquid results in profound hypoplastic changes in the lung [2]. Cl^-^ enters the basolateral membrane across the Na^+^,K^+^,2Cl^-^ cotransporter (NKCC1) while Na^+^ is actively extruded by the Na,K-ATPase. K^+^ channels recycle K^+^ at the basolateral side hyperpolarizing the membrane which represents the driving force for apical Cl^-^ extrusion. Therefore, fetal lung fluid secretion can be inhibited by blockers of NKCC like bumetanide or inhibiting the Na,K-ATPase by ouabain [3–5]. However, the transporter mediating the extrusion of Cl^-^ at the apical side of epithelial cells is unknown, yet evidence suggests an involvement of the cystic fibrosis transmembrane conductance regulator (CFTR) [6–8]. CFTR is a cyclic adenosine monophosphate (cAMP)-regulated Cl^-^ channel located at the apical membrane of epithelial cells in several tissues. CFTR expression is developmentally regulated and shows temporal and tissue-specific distribution during fetal lung growth with its highest expression in the first and second trimester of gestation and a gradual decline in the third trimester [9–11]. In the postnatal lung, CFTR expression is largely limited to cells of the submucosal glands and individual cells of the airway [12,13]. By contrast, a large majority of cells express CFTR during fetal lung development [9] and its expression is about 75-fold higher than in the adult lung [14]. Timings of high and low CFTR expression correspond to the developmental stages of the lung. In sheep, high Cftr expression was observed during the pseudoglandular stage whereas Cftr expression decreases during the late canalicular stage [14]. This expression pattern suggests a role of CFTR in fetal lung development which has yet to be determined. Evidence for this assumption comes from animal studies analyzing transient knock-out and over-expression of Cftr during fetal development. Cftr over-expression in the pseudoglandular stage accelerated epithelial cell proliferation resulting in an increased lung surface area, saccular density and an increased number of air spaces [7]. By contrast, a transient *in utero* Cftr knock-out led to a cystic fibrosis (CF)-like phenotype [8,15]. These studies showed that even small or transient alteration of Cftr expression in the developing lung had profound consequences for lung organogenesis.

It is known that fluid absorption through epithelial Na^+^ transport is under hormonal control since the epithelial Na^+^ channel (ENaC) is regulated by different hormones, like cortisol, aldosterone and epinephrine [16–18]. Little is known about the effect of hormones on CFTR. However, it is noteworthy that the fetal cortisol level rises in the same developmental stage of lung development in which CFTR expression declines. Therefore, the goal of our study was to determine the effect of glucocorticoids (GCs) on CFTR mRNA expression and channel activity in different pulmonary cell types, analyze the mode of action and thereby provide a possible link between the fetal CFTR expression pattern and the fetal cortisol levels.

## Materials and Methods

### Tissue preparation

All animal care and experimental procedures were approved by the institutional review board (Landesdirektion Leipzig, Permit Number: T36/13). Sprague-Dawley rats were bred at the Medical Experimental Center (MEZ) of Leipzig University. Animals were housed in rooms with a controlled temperature (22°C), humidity (55%) and 12 h light-dark cycle. Food and water were freely available. Rats were euthanized by carbon dioxide inhalation.

#### Fetal distal lung epithelial (FDLE) cell isolation and culture

FDLE cells, a model of respiratory cells in the late canalicular / early saccular stage of lung development, were isolated from lungs of 19–20 d gestation fetal rats as described previously [19,20]. In brief, lungs were minced and digested in a solution with 0.125% trypsin (Life Technologies, Darmstadt, Germany) and 0.4 mg/ml DNAse (# LS006333, CellSystems, Troisdorf, Germany) in Hanks’ Balanced Salt Solution (HBSS) (Life Technologies) for 10 min at 37°C. Digestion was stopped by adding Minimum Essential Eagle's Medium (MEM) (Life Technologies) containing 10% fetal bovine serum (FBS) (Biochrom, Berlin, Germany). Cells were collected, centrifuged (440 x g) and re-suspended in 15 ml MEM containing 0.1% collagenase (# LS004194, CellSystems) and DNAse for further digestion. The solution was incubated for 15 min at 37°C. Collagenase activity was stopped by adding 15 ml MEM containing 10% FBS. Cells were plated twice for 1 h to remove contaminating fibroblasts. The supernatant contained epithelial cells with > 95% purity [20]. For Ussing chamber measurements, cells were seeded on permeable Snapwell supports (Costar # 3407, Corning, Inc., NY, surface area 1 cm^2^) at a density of 10^6^ cells per insert. For real-time quantitative PCR (RT-qPCR) analyses, cells were seeded on permeable Transwell supports (Costar # 3412, surface area 4.5 cm^2^) at a density of 2x10^6^ cells per insert. FDLE cells were cultured under submerged conditions and medium was changed daily. The culture medium contained MEM with 10% FBS, 2 mM L-glutamine (Life Technologies), penicillin (100 U/ml, Life Technologies), streptomycin (100 μg/ml, Life Technologies) and amphotericin B (0.25 μg/ml, Life Technologies). For all analyzed cell types serum-free complete medium (Cellgro, Mediatech, Herndon, VA), supplemented with dexamethasone (Sigma-Aldrich, Taufkirchen, Germany) was added 24 h before measurement or as stated otherwise. Measurements of FDLE monolayers were performed 72–96 h after isolation. At this time point monolayers exhibited a high transepithelial resistance (R_te_) of 997.3 ± 424.2 Ω*cm^2^ (mean ± SD). Longer incubation times are not recommended since alveolar type II (ATII) cells have been described to transition into alveolar type I (ATI) cells after prolonged incubation *in vitro* [21]. To determine the involvement of the glucocorticoid receptor (GR), mifepristone (10 μM, # M-8046, Sigma-Aldrich) was used. Cells subjected to different experimental conditions were always age matched, derived from the same litter, treated equally and recorded simultaneously.

#### Isolation of adult ATII cells

A complete description can be found elsewhere [22]. In brief, Sprague-Dawley rats (140–200 g) were anesthetized (ketamin 10% and xylazil 2%), and injected with heparin (400 IU/kg). The lungs were perfused with saline solution and removed from the body. Resected lungs were washed repeatedly and incubated twice with a saline solution containing 0.25 mg/ml elastase (# EC134, Elastin Products Company Inc., MO, USA) and 0.05 mg/ml trypsin at 37°C for 15 min. Afterwards lung tissue was dissected from the bronchi and trachea and sliced into 1 mm bits within a DNase containing solution. Enzyme reaction was stopped by incubation with FBS (37°C, 2 min). The tissue was filtered 3 times through gauze and nylon meshes (mesh width: 120, 20, and 10 μm) and the filtrate centrifuged for 8 min at 130 x g. After re-suspending in Dulbecco’s Modified Eagle’s Medium (DMEM) (Life Technologies), cells were incubated on IgG (Life Technologies) coated plastic dishes at 37°C for 15 min. Non-adhering cells were again centrifuged for 8 min at 130 x g and re-suspended in DMEM with 10% FBS, penicillin (100 U/ml) and streptomycin (100 μg/ml). Cells were seeded according to the FDLE cells, and measurements and RNA isolation performed 96 h after isolation. Their mean R_te_ was 814.1 ± 422.3 Ω*cm^2^.

#### Isolation of primary airway epithelial cells

Isolation of primary airway epithelial cells has been described previously [23]. Briefly, the trachea proximal to the bronchial bifurcation was isolated from euthanized male Sprague-Dawley rats (140–200 g). Esophageal remnants and adherent adipose tissue were removed. The trachea was opened longitudinally and rinsed with DMEM before incubation in DMEM with 0.1% protease XIV (# P5147, Sigma-Aldrich), 0.01% DNase and 1% FBS for 21 h at 4°C. The digestion was stopped by the addition of 1 Vol. FBS. The trachea was then agitated and scraped with a cell scraper to detach the airway epithelial cells. The obtained cell suspension was centrifuged twice at 500 x g for 5 min and re-suspended in cell culture media. Cell culture media consisted of DMEM-F12 (Life Technologies) with 1 μg/ml insulin (# 91077C, Sigma-Aldrich), 7.5 μg/ml transferrin (# 354204, Corning), 1 μM hydrocortisone (# H0888, Sigma-Aldich), 30 nM 3,5,3’-triiodothyronine (# T6397, Sigma-Aldrich), 2.5 ng/ml epidermal growth factor (# 354052, Corning), 10 ng/ml endothelial cell growth supplement (# 354006, Corning), penicillin (100 U/ml), streptomycin (100 μg/ml) and amphotericin B (0.25 μg/ml) which was supplemented (1:1) with 3T3 fibroblast (from ATCC, # CCL-92)-conditioned DMEM containing 2% FBS. Cells were seeded on collagen-coated permeable supports at a density of 2x10^5^ cells per Snapwell insert and 4x10^5^ cells per Transwell insert. Measurements and RNA isolation were done approximately 10 days after plating, when R_te_ reached values >300 Ω*cm^2^ (mean R_te_: 2568 ± 2097 Ω*cm^2^).

### Culture of cell lines

A549 cells (from ATCC, # CCL-185), an adenocarcinoma-derived human alveolar epithelial cell line, and Calu-3 cells (from ATCC, # HTB-55), derived from human bronchial submucosal glands, were kindly provided by Dr. Getu Abraham (Institute of Pharmacology, Pharmacy and Toxicology, Faculty of Veterinary Medicine, University of Leipzig). A549 cells (passage 10–26) were cultured in DMEM with 10% FBS and Calu-3 cells (passage 23–30) in DMEM-F12 with 10% FBS, penicillin (100 U/ml), streptomycin (100 μg/ml) and 1% non-essential amino acids (Life Technologies), and passaged 1–2 times weekly. A549 cells were seeded on Transwell supports at a density of 2x10^6^ cells per insert according to the FDLE cells and RNA was isolated after 5 days. Calu-3 cells were seeded at a density of 5x10^5^ per Snapwell insert and 1x10^6^ per Transwell insert. After 10 days, Calu-3 cells were subjected to air-liquid interface conditions and R_te_ was measured every two days during medium change with an EVOM epithelial voltohmmeter (World Precision Instruments, Sarasota, FL, USA) with STX-2 chopstick electrodes. Measurements and RNA isolation were done approximately 14–21 days after plating of Calu-3 cells on permeable supports, when R_te_ reached values >300 Ω*cm^2^ (mean R_te_: 888.2 ± 400.8 Ω*cm^2^). The 16HBE14ο- cell line (passage 2.82–86) was generated from human bronchial surface epithelium as described previously [24] and was kindly provided by Dr. Dieter Gruenert (Mt Zion Research Center, University of California, San Francisco, CA, USA). The 16HBE14o- cells were grown in MEM with 10% FBS, 2 mM L-glutamine, penicillin (100 U/ml) and streptomycin (100 μg/ml). Cell culture flasks and permeable supports were coated with a solution containing bovine collagen I (Corning), human fibronectin (Corning) and bovine serum albumin (Sigma-Aldrich) in LHC basal medium (Life Technologies). Cells were seeded at a density of 5x10^5^ per Transwell insert. After 7 days, 16HBE14o^-^ cells were subjected to air-liquid interface conditions and after 14 days RNA isolation was performed.

To determine the involvement of the phosphoinositide 3-kinase (PI3K), the inhibitor LY-294002 (10 μM, # 1130 TOCRIS Bioscience, Bristol, UK) was used.

### Measurement of mRNA expression

Total RNA was isolated using the RNeasy Kit (Qiagen, Hilden, Germany) and treated with DNase I (Life Technologies) according to the manufacturer’s instructions. Reverse transcription was carried out in two steps by first pre-annealing of 1 μg RNA with Oligo(dT)_18_ primers (ThermoFisher Scientific, St. Leon-Rot, Germany), followed by the addition of Superscript III (Life Technologies) and incubation at 55°C for 1 h and 75°C for 15 min. The resulting cDNA was diluted 1:10 in Tris-EDTA buffer (AppliChem, Darmstadt, Germany) and the Platinum Taq polymerase (Life Technologies) was used for RT-qPCR, following the manufacturer’s instructions. Reactions were conducted with the CFX 96 Real-Time system (BioRad, Munich, Germany) with SYBR-Green (Molecular Probes, Eugene Oregon, USA). Transcripts of target genes were amplified using the gene-specific primers listed in Table 1. Absolute quantification was performed using a several fold dilution of target specific plasmid DNA as internal standard curve. Amplification efficiencies were close to 100%, calculated by standard curve analysis and standard curve plots showed a high coefficient of determination (R^2^>0.99). The resulting molecule concentrations were normalized to a reference gene (Mrps18a: mitochondrial ribosomal protein S18a). Constant expression of Mrps18a was confirmed against other common reference genes. The fold change of mRNA levels was calculated with the relative standard curve method. Measurements were performed in technical triplicates and at least 3 biological replicates. Melting curves and gel electrophoresis of PCR products were routinely performed to determine the specificity of the PCR reaction.

**Table 1:** Gene-specific primers.

gene	forward	reverse
CFTR-humanNM_000492.3	GGGCTGTGTCCTAAGCCATGGCCA	GATGGCTTGCCGGAAGAGGCTCC
Cftr-ratNM_031506.1	GCCTTCGCTGGTTGCACAGTAGTC	GCTTCTCCAGCACCCAGCACTAGA
MRPS18A-humanNM_001193343.1	CGGCTTCCAGCTCGCGGGTTC	GGTACCTGCTCGGTGGGCCATC
Mrps18a-ratNM_198756.1	GCGACCGGCTGGTTATGGCT	GGGCACTGGCCTGAGGGATTAG
ANO1-humanNM_018043.5	CGGAAACAGATGCGACTCAAC	TGATCCTTGACAGCCTCCTCTT
Ano1-ratNM_001107564.1	CCTGTTCGTTGCGTCCTTCCCTC	GAGCGTGTGGTTGACGAAGCCG
ANO6-human (a-d)NM_001025356.2NM_001142678.1NM_001142679.1NM_001204803.1	CAGCCACCAGAAGCCGCATTG	CTCTGACTGACGGCGGAATTTGC
Ano6-ratNM_001108108.1	GGGACCCGGTGTACTGGCTGG	CATAGAACAATCCCAGCCTGCCC
CLC5-V2/5-humanNM_001127898.3NM_001272102.1	GCCCCGAGTTTGGGGCTTTA	CCCTGCTGAAAGCCTCTGTTATCC
Clc5-ratNM_017106.1	CGTGGCTTGCTGCTGTGGGAA	GAGAGCAGCGAAGAAGGAACGCC
NKCC1-humanNM_001256461.1	GAATCCAAAGGCCCTATTGTGCC	GCCATCGCTCTCCGGTCATG
Nkcc1-ratNM_031798.1	GGCCATCGCTGACTTCGTCATAGG	GCAAGGTCACCCGAGATGTTCGC

### Electrophysiological measurements

A detailed description of Ussing chamber measurement procedures is reported elsewhere [19]. Experiments were included in the data analyses only when R_te_ exceeded 300 Ω*cm^2^ throughout the measurement. Ussing chambers were filled with a ringer solution containing: Na^+^ 145 mM, K^+^ 5 mM, Ca^2+^ 1.2 mM, Mg^2+^ 1.2 mM, Cl^–^ 125 mM, HCO_3_^–^ 25 mM, H_2_PO_4_^-^ 3.3 mM, HPO_4_^2-^ 0.8 mM (pH 7.4). The basolateral side contained 10 mM glucose whereas 10 mM mannitol was used in the apical compartment. Equivalent short-circuit currents (I_SC_) were assessed every 20 s by measuring transepithelial voltage (V_te_) and R_te_ using a transepithelial current clamp (Physiologic Instruments, San Diego, CA), and calculating the quotient I_SC_ = V_te_/R_te_. Amiloride (10 μM, # A-7410, Sigma-Aldrich), an inhibitor of ENaC, was added to the apical compartment to inhibit amiloride-sensitive Na^+^ channels. Forskolin (10 μM, # F-6886, Sigma-Aldrich) was added to the apical compartment to increase the intracellular cAMP concentration and thereby activate cAMP-sensitive ion channels like CFTR. Finally, glibenclamide (200 μM, # G-0639, Sigma-Aldrich) or CFTR_inh_172 (10 μM, # 3430, TOCRIS Bioscience) was applied apically to determine the glibenclamide-sensitive or CFTR_inh_172-sensitive I_SC_, a measure of CFTR activity. In another experiment measurements were carried out in Cl^-^-free solution containing: Na^+^ 145 mM, K^+^ 5 mM, Ca^2+^ 4 mM, Mg^2+^ 1 mM, gluconate 125 mM, HCO_3_^-^ 25 mM, H_2_PO_4_^-^ 3.3 mM, HPO_4_^2-^ 0.8 mM (pH 7.4) or HCO_3_^-^-free solution containing: Na^+^ 145 mM, K^+^ 5 mM, Ca^2+^ 1.2 mM, Mg^2+^ 1.2 mM, Cl^-^ 145 mM, H_2_PO_4_^-^ 3.3 mM, HPO_4_^2-^ 0.8 mM (pH 7.4). In HCO_3_^-^-free measurements Ussing chambers were gassed with 100% O_2_ instead of 95% O_2_ / 5% CO_2_.

Amiloride was dissolved in water; forskolin, glibenclamide, CFTR_inh_172, mifepristone and LY-294002 were prepared in DMSO and dexamethasone in 100% ethanol. Control monolayers were treated with the respective solvent to exclude solvent influence on the evoked responses.

### Statistical analysis

For statistical analyses, GraphPad Prism (version 5.03; GraphPad Software, Inc, San Diego, CA, USA) was used. Differences among groups treated with dexamethasone and controls were evaluated by unpaired T-test, or analysis of variance (ANOVA) followed by Dunnett’s or Tukey’s *post hoc* test, as appropriate. A probability of p<0.05 was considered significant for all statistical analyses.

## Results

### Effect of dexamethasone on CFTR/Cftr expression and activity in alveolar cells

**Fig 1:**
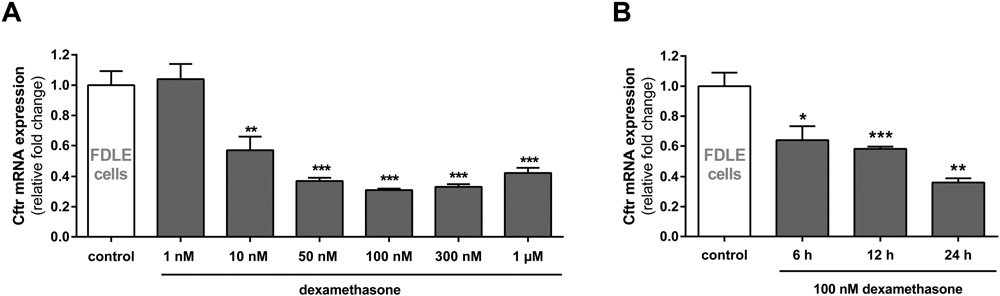
Dexamethasone reduces Cftr mRNA expression in FDLE cells. Graphs represent the mean + SEM for normalized Cftr mRNA expression acquired by RT-qPCR. **A:** Dose-response curve of dexamethasone effect (1 nM–1 μM for 24 h, n = 4, ** p<0.01; *** p<0.001, ANOVA with Dunnett's *post hoc* test compared to control monolayers without dexamethasone addition). **B:** Time course of Cftr mRNA expression in response to 100 nM dexamethasone for 6, 12 and 24 h (n = 4, * p<0.05; ** p<0.01; *** p<0.001, T-test).

Cftr mRNA expression was determined in FDLE cells incubated in medium containing different dexamethasone concentrations (1 nM, 10 nM, 50 nM, 100 nM, 300 nM and 1 μM for 24 h) and compared to control monolayers without hormone addition. Cftr mRNA expression decreased with increasing dexamethasone concentrations in a dose-dependent manner, starting at 10 nM dexamethasone (Fig 1A). At 100 nM dexamethasone, Cftr mRNA expression was maximally inhibited, with mRNA expression levels decreased by 70% (p<0.001). The time course of the dexamethasone modulation was determined at different time points (6, 12 and 24 h) using 100 nM dexamethasone demonstrating a reduction of Cftr mRNA expression within 6 h (p<0.05) that further decreased thereafter (Fig 1B).

**Fig 2:**
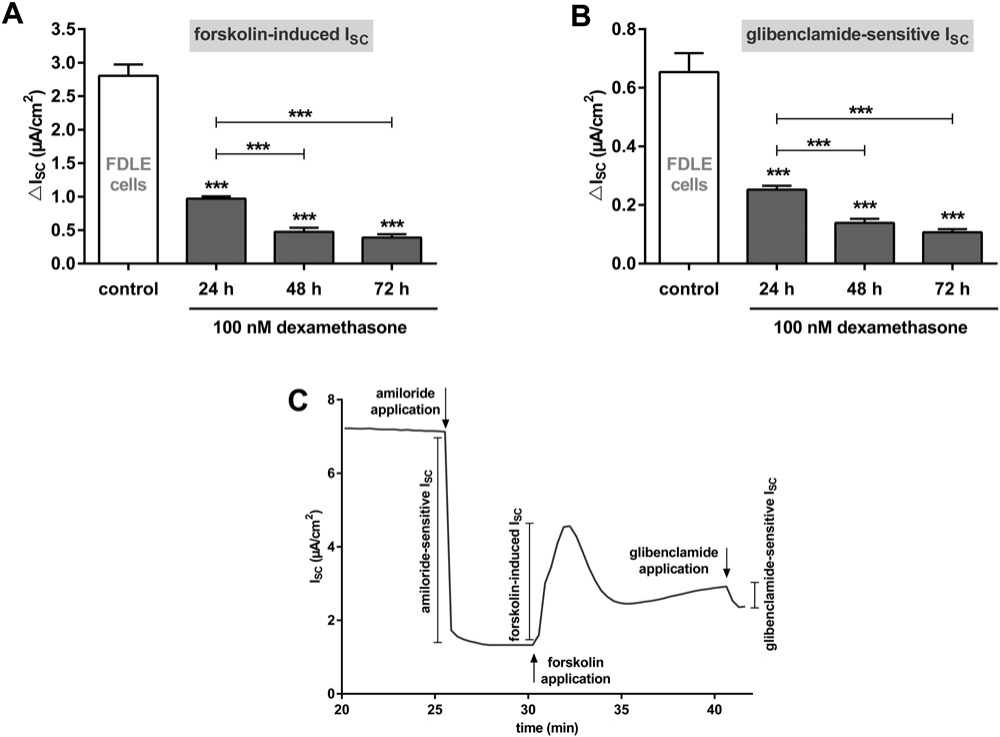
Dexamethasone reduces Cftr channel activity in FDLE cells. Graphs represent the mean + SEM of I_SC_ in response to 100 nM dexamethasone for 24, 48 and 72 h measured in Ussing chambers. **A:** Forskolin-induced I_SC_ (n = 25–56, *** p<0.001, ANOVA with Tukey's *post hoc* test). **B:** Glibenclamide-sensitive I_SC_ (n = 25–55, *** p<0.001, ANOVA with Tukey's *post hoc* test). **C:** Typical current tracing of FDLE monolayers.

Next, we determined the effect of 100 nM dexamethasone on Cftr channel activity in Ussing chambers. Following FDLE cell isolation, cell culture media was changed to serum-free medium after 24 h and monolayer were divided into four groups. Control monolayers were cultured in serum-free medium for 72 h, whereas the other groups were supplemented with dexamethasone for either, 24 h, 48 h or 72 h. Ussing chamber measurements were performed 96 h after cell isolation. After the basal current reached a plateau, amiloride was applied to inhibit amiloride-sensitive Na^+^ channels like ENaC, followed by the addition of forskolin to maximally stimulate existing Cftr channels alone without interference from ENaC. Finally, glibenclamide was added to inhibit Cftr activity (Fig 2C). After calculating the forskolin-induced and glibenclamide-sensitive I_SC_, a measure of CFTR activity, dexamethasone-stimulated FDLE monolayers were compared to unstimulated control monolayers. FDLE monolayers treated with dexamethasone for 24 h showed a decreased Cftr activity by more than 65%, as shown by the significantly reduced forskolin-induced and glibenclamide-sensitive I_SC_ (p<0.001, Fig 2A and 2B). Prolonged dexamethasone treatment for 48 and 72 h further decreased the forskolin-induced and glibenclamide-sensitive I_SC_ (p<0.001, Fig 2A and 2B).

**Fig 3:**
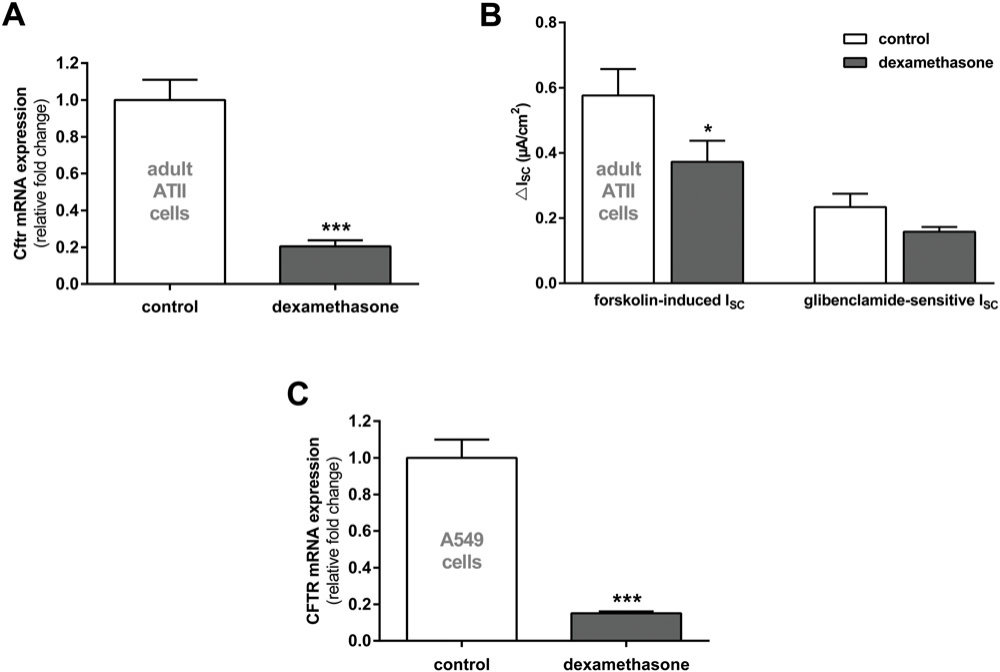
Dexamethasone reduces CFTR/Cftr mRNA expression and channel activity in adult alveolar cells. **A/B:** primary adult ATII cells. **A:** Graph represents the mean + SEM for normalized Cftr mRNA expression in response to 100 nM dexamethasone for 24 h acquired by RT-qPCR (n = 6, *** p<0.001 by T-test compared to control monolayers without dexamethasone addition). **B:** Graph represents the mean + SEM of I_SC_ in response to 100 nM dexamethasone for 24 h measured in Ussing chambers. Forskolin-induced I_SC_ (n = 29–31, * p<0.05 by T-test compared to control monolayers without dexamethasone addition) and glibenclamide-sensitive I_SC_ (n = 27–28). **C:** A549 cells. Graph represents the mean + SEM for normalized CFTR mRNA expression in response to 100 nM dexamethasone for 24 h acquired by RT-qPCR (n = 8, *** p<0.001, T-test compared to control cells without dexamethasone addition).

Since FDLE cells represent a fetal primary cell culture, primary adult ATII cells were analyzed to examine if the observed effects were dependent on the developmental stage of the cells. 100 nM dexamethasone also significantly reduced Cftr mRNA expression in adult ATII cells (p<0.001, Fig 3A). Furthermore, Cftr channel activity measured as forskolin-induced I_SC_ in monolayers treated with 100 nM dexamethasone for 24 h was significantly reduced (p<0.05, Fig 3B), and the glibenclamide-sensitive I_SC_ demonstrated a non-significant trend in the same direction. To determine whether the analyzed effect of dexamethasone on Cftr in alveolar cells was species-specific the analysis of CFTR mRNA expression was repeated with the human alveolar cell line A549. Results showed that 100 nM dexamethasone for 24 h also reduced CFTR mRNA expression in human A549 cells by 80% (p<0.001, Fig 3C).

**Fig 4:**
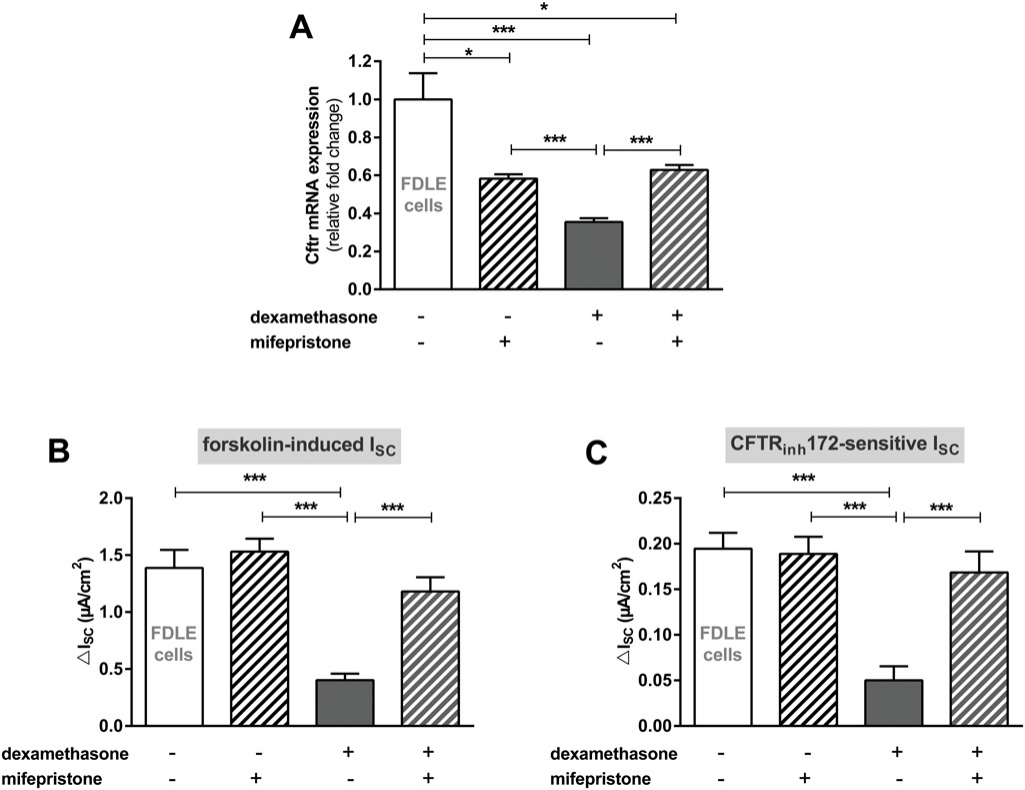
Mifepristone restores Cftr mRNA expression and channel activity in the presence of dexamethasone in FDLE cells. **A:** Graph represents the mean + SEM for normalized Cftr mRNA expression in response to 100 nM dexamethasone and mifepristone for 24 h acquired by RT-qPCR (n = 4, * p<0.05, *** p<0.001, ANOVA with Tukey's *post hoc* test). **B/C:** Graphs represent the mean + SEM of I_SC_ in response to 100 nM dexamethasone and mifepristone for 24 h measured in Ussing chambers. **B:** Forskolin-induced I_SC_ (n = 10–17, *** p<0.001, ANOVA with Tukey's *post hoc* test). **C:** CFTR_inh_172-sensitive I_SC_ (n = 8–13, *** p<0.001, ANOVA with Tukey's *post hoc* test).

To determine the pathway of dexamethasone action we used mifepristone to block the GR. Inhibition by mifepristone restored Cftr mRNA expression and channel activity in FDLE cells (Fig 4). Cftr mRNA expression analysis showed that mifepristone by itself reduced Cftr expression (p<0.05, Fig 4A) yet addition of dexamethasone did not further decrease Cftr expression in the presence of mifepristone. By contrast, Cftr expression in the sole presence of dexamethasone was significantly lower than in mifepristone- or mifepristone/dexamethasone-incubated FDLE cells (p<0.001). Cftr activity in dexamethasone-incubated FDLE monolayers was restored to control levels by mifepristone and mifepristone by itself did not affect Cftr activity as shown for the forskolin-induced and CFTR_172_inh-sensitive I_SC_ in Ussing chambers (p<0.001, Fig 4B and 4C).

### Effect of dexamethasone on CFTR/Cftr expression and activity in airway-derived cells

**Fig 5:**
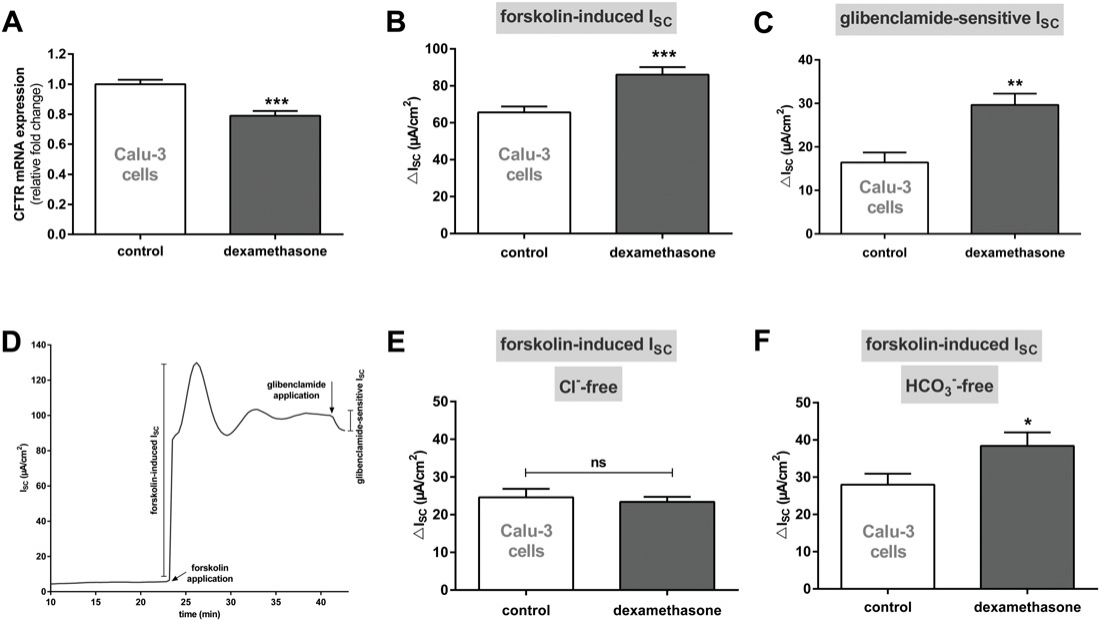
Dexamethasone reduces CFTR mRNA expression and increases channel activity in Calu-3 cells. **A:** Graph represents the mean + SEM for normalized CFTR mRNA expression in response to 100 nM dexamethasone for 24 h acquired by RT-qPCR (n = 6, *** p<0.001, T-test compared to control cells without dexamethasone addition). **B/C/E/F:** Graphs represent the mean + SEM of I_SC_ in response to 100 nM dexamethasone for 24 h measured in Ussing chambers. **B:** Forskolin-induced I_SC_ (n = 12, *** p<0.001, T-test compared to control cells without dexamethasone addition). **C:** Glibenclamide-sensitive I_SC_ (n = 6, ** p<0.01, T-test compared to control cells without dexamethasone addition). **D:** Typical current tracing of Calu-3 cells. **E:** Forskolin-induced I_SC_ measured in Cl^-^-free solution (n = 14–16, ns = not significant). **F:** Forskolin-induced I_SC_ measured in HCO_3_^-^-free solution (n = 16, * p<0.05, T-test compared to control cells without dexamethasone addition).

Next, we sought to determine whether the effect of dexamethasone on CFTR was related to the cell type. Therefore, we analyzed the effect of 100 nM dexamethasone for 24 h in human bronchial submucosal gland-derived Calu-3 cells. Similar to alveolar cells, treatment of Calu-3 cells with dexamethasone led to a significant reduction of CFTR mRNA expression (p<0.001, Fig 5A). However, the extent by which the CFTR expression level was reduced differed between alveolar and bronchial cells. In Calu-3 cells mRNA expression was reduced by approximately 20% compared to control cells whereas in alveolar cells CFTR/Cftr expression diminished by 70–80%. By contrast, CFTR channel activity, as measured in Ussing chambers, was actually increased by 100 nM dexamethasone. As shown in Fig 5B and 5C, treatment of Calu-3 cells with dexamethasone led to a significantly increased forskolin-induced and glibenclamide-sensitive I_SC_ (p<0.01, p<0.001). Since CFTR is permeable for both Cl^-^ or HCO_3_^-^ ions, we repeated the measurements in solutions either without Cl^-^ or HCO_3_^-^. Omitting Cl^-^ abolished the effect of dexamethasone on the forskolin-induced I_SC_ (Fig 5E). By contrast, omitting HCO_3_^-^ did not affect the stimulation of forskolin-induced I_SC_ by dexamethasone which was significantly higher compared to control cells (p<0.05, Fig 5F).

**Fig 6:**
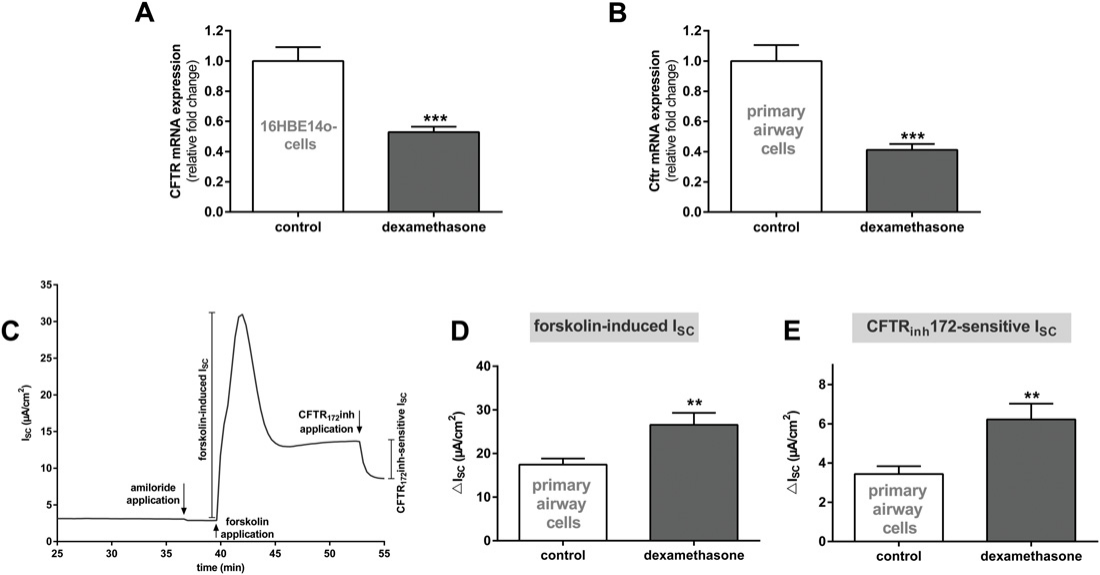
Dexamethasone reduces CFTR/Cftr mRNA expression and increases channel activity in airway epithelial cells. **A/B:** Graphs represent the mean + SEM for normalized CFTR/Cftr mRNA expression in response to 100 nM dexamethasone for 24 h acquired by RT-qPCR. **A:** 16HBE14o- cells (n = 8, *** p<0.001, T-test compared to control cells without dexamethasone addition). **B:** Primary rat airway epithelial cells (n = 7–8, *** p<0.001, T-test compared to control cells without dexamethasone addition). **C:** Typical current tracing of primary airway epithelial cells. D/E: Graphs represent the mean + SEM of I_SC_ in response to 100 nM dexamethasone for 24 h measured in Ussing chambers. **D:** Forskolin-induced I_SC_ (n = 20–23, ** p<0.01, T-test compared to control cells without dexamethasone addition). E: CFTR_172_inh-sensitive I_SC_ (n = 19–20, ** p<0.01, T-test compared to control cells without dexamethasone addition).

Furthermore, experiments were repeated with human bronchial 16HBE14o- cells incubated with 100 nM dexamethasone for 24 h. In accordance, CFTR mRNA expression was reduced by 50% in the presence of dexamethasone (p<0.001, Fig 6A). Since Calu-3 and 16HBE14o- cells represent immortalized cell lines we further analyzed the effect of dexamethasone on Cftr expression and activity in primary rat airway epithelial cells. Likewise, Cftr mRNA expression was reduced by 100 nM dexamethasone for 24 h in primary airway epithelial cells (p<0.001, Fig 6B). CFTR activity was increased as demonstrated by the significantly elevated forskolin-induced and CFTR_172_inh-sensitive I_SC_ in primary airway epithelial cells (p<0.01, Fig 6D and 6E). Therefore, results obtained with Calu-3 and 16HBE14o- cells were confirmed by primary airway epithelial cells.

**Fig 7:**
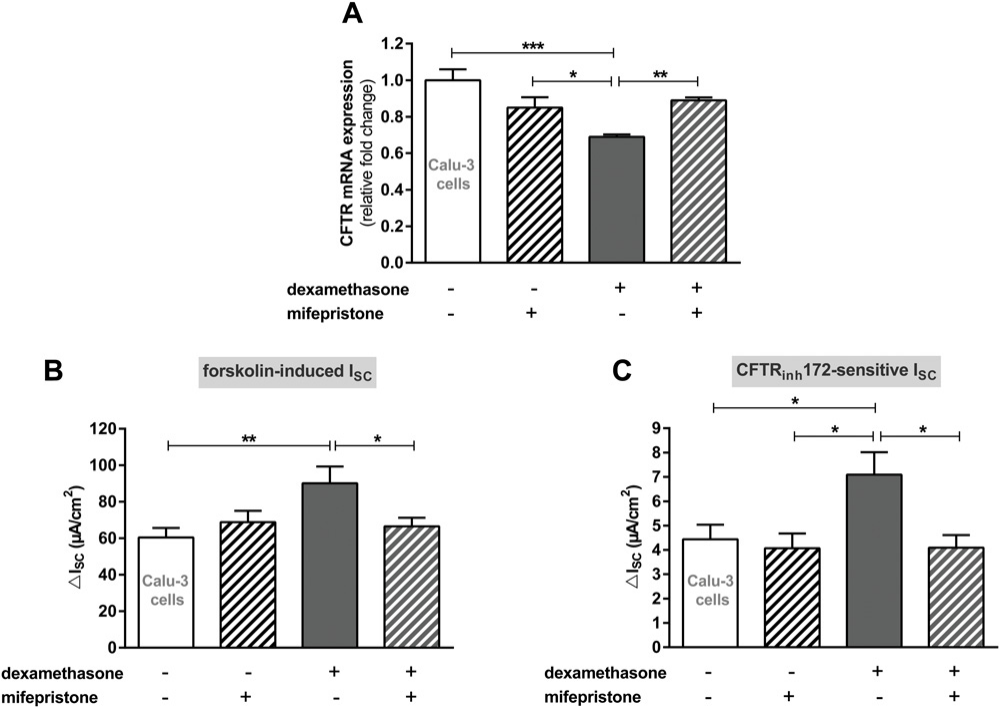
Mifepristone restores CFTR mRNA expression and reduces channel activity in the presence of dexamethasone in Calu-3 cells. **A:** Graph represents the mean + SEM for normalized CFTR mRNA expression in response to 100 nM dexamethasone and mifepristone for 24 h acquired by RT-qPCR (n = 3–4, * p<0.05, ** p<0.01, *** p<0.001, ANOVA with Tukey's *post hoc* test). **B/C:** Graphs represent the mean + SEM of I_SC_ in response to 100 nM dexamethasone and mifepristone for 24 h measured in Ussing chambers. **B:** Forskolin-induced I_SC_ (n = 7–12, * p<0.05, ** p<0.01, ANOVA with Tukey's *post hoc* test). **C:** CFTR_inh_172-sensitive I_SC_ (n = 7–12, * p<0.05, ANOVA with Tukey's *post hoc* test).

Measurements using Calu-3 cells were also performed in the presence of mifepristone to address the function of the GR in these cells. Mifepristone restored CFTR mRNA expression because no difference was observed between mifepristone- and mifepristone/dexamethasone-incubated Calu-3 cells (Fig 7A). In addition, the dexamethasone-induced increase of forskolin-induced and CFTR_172_inh-sensitive I_SC_ measured in Ussing chambers was indistinguishable from measurements without dexamethasone when mifepristone was present (Fig 7B and 7C).

**Fig 8:**
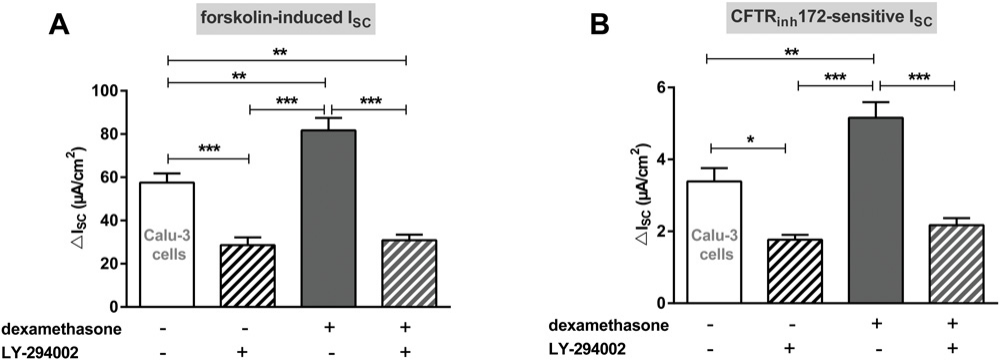
LY-294002 prevents the increase of CFTR activity induced by dexamethasone in Calu-3 cells. Graphs represent the mean + SEM of I_SC_ in response to 100 nM dexamethasone and LY-294002 for 24 h measured in Ussing chambers. **A:** Forskolin-induced I_SC_ (n = 12–18, ** p<0.01, *** p<0.001, ANOVA with Tukey's *post hoc* test). **B:** CFTR_inh_172-sensitive I_SC_ (n = 12–18, * p<0.05, ** p<0.01, *** p<0.001, ANOVA with Tukey's *post hoc* test).

Since dexamethasone reduces CFTR mRNA expression in Calu-3 cells, the increase of CFTR activity has to be achieved by post-translational mechanisms. Because kinases are known to influence the activity of ion channels, we determined whether the PI3K is involved. Inhibiting the PI3K with LY-294002 decreased CFTR activity in control and dexamethasone-stimulated cells (p<0.05, p<0.01, p<0.001, Fig 8A and 8B). Furthermore, dexamethasone was unable to increase CFTR activity when LY-294002 was present.

### Effect of dexamethasone on mRNA expression of alternative chloride transporters

**Fig 9:**
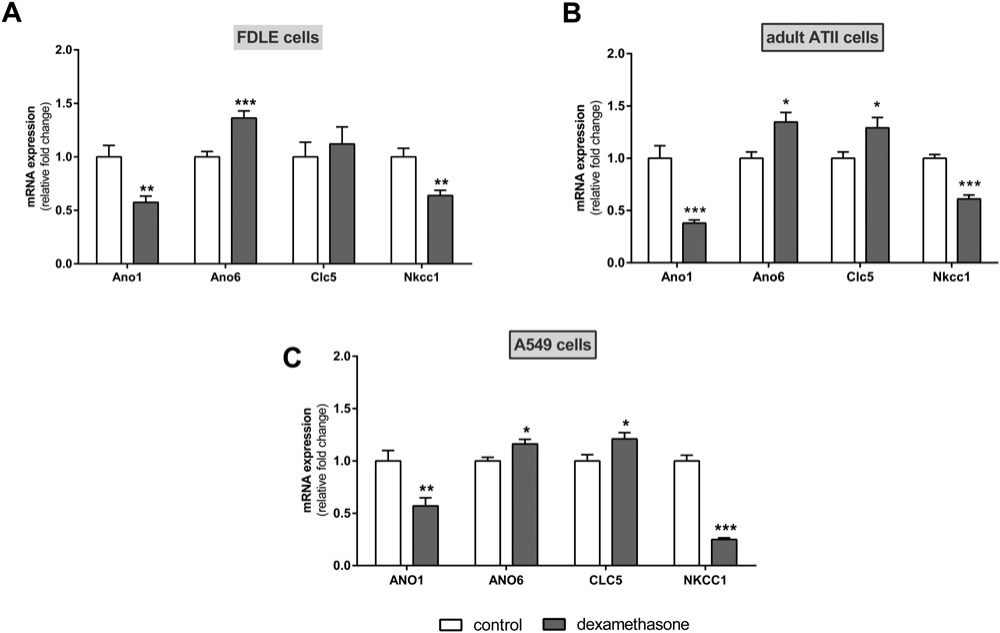
Dexamethasone modulates alternative chloride transporters in alveolar cells. Graphs represent the mean + SEM for normalized mRNA expression in response to 100 nM dexamethasone for 24 h acquired by RT-qPCR. **A:** FDLE cell monolayers (n = 7–12, ** p<0.01, *** p<0.001, T-test compared to control monolayers without dexamethasone addition). **B:** Adult ATII cells (n = 4–6, * p<0.05, *** p<0.001, T-test compared to control monolayers without dexamethasone addition). **C:** A549 cells (n = 8–12, * p<0.05, ** p<0.01, *** p<0.001, T-test compared to control cells without dexamethasone addition).

**Fig 10:**
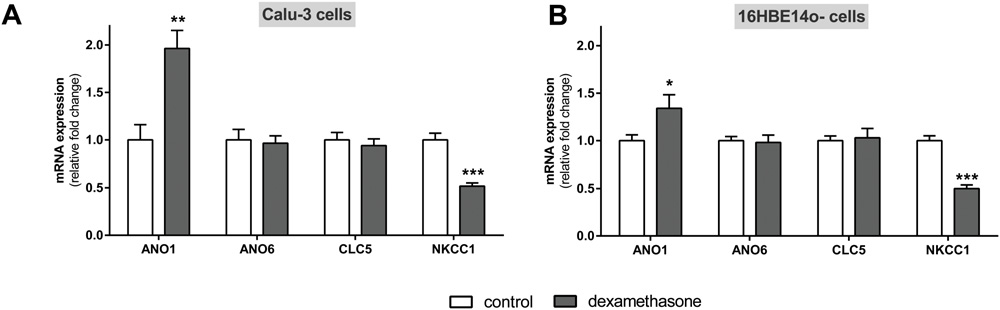
Dexamethasone modulates alternative chloride transporters in bronchial cells. Graphs represent the mean + SEM for normalized mRNA expression in response to 100 nM dexamethasone for 24 h acquired by RT-qPCR. **A:** Calu-3 cells (n = 8–14, ** p<0.01, *** p<0.001, T-test compared to control cells without dexamethasone addition). **B:** 16HBE14o- cells (n = 7–8, * p<0.05, *** p<0.001, T-test compared to control cells without dexamethasone addition).

Finally, we determined the effect of dexamethasone on alternative Cl^-^ transporters in alveolar (Fig 9) and bronchial cells (Fig 10). Anoctamine 1 [ANO1/Ano1, TMEM16A] mRNA expression was decreased by dexamethasone in all analyzed alveolar cell types (p<0.01, p<0.001, Fig 9A–9C) and increased in bronchial Calu-3 and 16HBE14o- cells (p<0.05, p<0.01, Fig 10A and 10B). Furthermore, anoctamin 6 [ANO6/Ano6, TMEM16F] and voltage-gated chloride channel 5 [CLC5/Clc5] mRNA expression were increased by dexamethasone in alveolar cells (p<0.05, p<0.001, Fig 9A–9C) yet no effect was seen in Calu-3 and 16HBE14o- cells (Fig 10A and 10B). NKCC1/Nkcc1 mRNA expression was decreased by dexamethasone in alveolar and also in bronchial cells alike (p<0.01, p<0.001, Figs 9A–9C; 10A and 10B).

## Discussion

The study shows that dexamethasone reduces CFTR/Cftr mRNA expression in a concentration dependent manner. The strongest inhibition was achieved with 100 nM dexamethasone, which reduced the Cftr expression level by 70%. Dexamethasone concentrations above 100 nM did not further decrease Cftr expression. Noteworthy, a lower effect of higher dexamethasone concentrations on ENaC mRNA expression has been described before [25]. The response to GC treatment occurred within hours of exposure and a down-regulation of Cftr expression was detected only 6 h after dexamethasone addition. Cftr expression was further reduced after 24 h exposure. The same response was seen for the Cftr activity in Ussing chamber measurements in which the forskolin-induced and glibenclamide-sensitive I_SC_ were reduced by 65% within 24 h of dexamethasone exposure. Cftr activity further decreased with prolonged dexamethasone treatment reaching a reduction of almost 90% after 72 h.

As shown previously, Cftr expression is developmentally regulated and is greatest during the first and second trimesters [14]. During the canalicular stage of lung development when differentiation into ATI and ATII cells occurs, Cftr expression begins to gradually decline and remains low until birth [14]. Fetal cortisol levels start to rise at the 20–24^th^ week of gestation (canalicular stage) and gradually increase thereafter with a surge prior to labor [26]. In our experiments with FDLE cells, Cftr activity is rather small, however FDLE cells are differentiated ATII cells from the late canalicular / early saccular stage of lung development, when a decline of Cftr presumably has already occurred in the fetus and Cftr activity is still strongly responsive to GC exposure. We therefore propose that the rise of fetal cortisol levels during the late canalicular stage of lung development is responsible for the decline of CFTR expression observed during this stage. In adult rat ATII cells Cftr mRNA expression and activity were affected in the same manner as in FDLE cells. Therefore, the effect of GCs does not depend on the developmental stage of the cells. In human alveolar A549 cells, dexamethasone also reduced CFTR mRNA expression by more than 80% showing that the effect of GCs is not species-specific as human and rodent alveolar cells reacted similarly. Taken together, GCs strongly antagonize CFTR/Cftr expression and activity in cells of alveolar origin, which possibly represents an important mechanism in the transition from fluid secretion to absorption in fetal lung development. It is known that GCs stimulate absorption by increasing the Na^+^ transport via ENaC [17,18]; however, the notion that GCs also reduce secretion by inhibiting CFTR has not been suggested before.

Studies addressing the stimulation of lung growth possibly by enhancing epithelial Cl^-^ secretion could be valuable in circumstances associated with pulmonary hypoplasia as congenital diaphragmatic hernia (CDH) or prolonged oligohydramnios [27]. Therefore, knowledge of the hormonal regulation of pulmonary Cl^-^ secretion is an important step in understanding and possibly influencing fetal lung growth. A line of evidence suggests that CFTR is indeed involved in prenatal Cl^-^ secretion. First of all the expression pattern of CFTR suggests an involvement in fetal lung development. In fact, it has been shown that Cftr over-expression during lung development increases epithelial cell proliferation and enhances secretory cell differentiation [6]. Criticism was raised against this hypothesis due to the lack of overt pathological changes in the CF fetus, however data suggests that early functional changes are present in the lungs of CF patients despite the lack of observable clinical pathology [28–31]. CDH lungs exhibit epithelial cell immaturity supposedly resulting from an impaired branching morphogenesis in early lung development [32]. In a nitrofen-treated CDH rat model, Larson and Cohen, 2006 analyzed whether Cftr over-expression during fetal development would improve the phenotype. The treatment enhanced internal surface area, saccular density, saccular number and amount of saccular air spaces in the lungs suggesting an acceleration of lung development by Cftr over-expression in the CDH model [7]. It was suggested that this effect was achieved by enhancing lung fluid secretion and consecutively the tension of lung tissue [33]. These studies therefore suggest that CFTR is involved in prenatal Cl^-^ secretion and thereby lung proliferation.

Near term, the rate of lung fluid secretion and the volume of fetal lung fluid decrease [27,34]. A slowing of fluid production in response to cortisol was reported in fetal guinea pigs, which occurred in addition to an increase of fluid absorption [35]. GCs are further implicated to inhibit lung growth in favor of differentiation [36]. More precisely, cortisol decreases fetal pulmonary cellular growth in early gestation whilst enhancing maturation and slowing growth as term approaches [37]. In fetal lung explants of the pseudoglandular stage it was demonstrated that treatment with dexamethasone distorted the overall branching pattern and resulted in a decreased proliferation of distal epithelial cells [38]. Furthermore, lung specific knock-out of the GR in mice results in a marked hypercellularity [39]. The morphological changes were the result of continued cell division in the distal and proximal epithelia and were attributed to an increased cell proliferation [39]. This suggests that if the lung is not responsive to GCs the epithelial proliferation proceeds unimpeded. It is known that growth arrest is a prerequisite to the induction for terminal differentiation of cells [40] which is mediated by GCs in the lung. Therefore the developmental effects of GCs on fetal lung growth are well known, but that this effect might be mediated by a modulation of CFTR expression has not yet been suggested. Another physiological function of the CFTR decline during fetal lung development could be to enable ENaC activity since CFTR is known to inhibit ENaC [41]. Moreover, ENaC mRNA expression is inversely proportional to the levels of Cftr mRNA in the developing lung suggesting a coordinated regulation [14]. Thus a decrease of CFTR would diminish ENaC inhibition and thereby further increase ENaC mediated Na^+^ absorption and perinatal fluid clearance in alveolar cells.

Since dexamethasone reduced CFTR/Cftr mRNA expression in alveolar cells it is surprising that inhibition of the GR by mifepristone alone also reduced Cftr expression. On the other hand, dexamethasone did not further decrease Cftr expression when mifepristone was present in the culture media. Therefore inhibition of the GR and incubation with dexamethasone both decrease Cftr expression. It appears that a minimal function of the GR may be necessary to optimize Cftr expression. Besides, mifepristone is also known to inhibit the progesterone receptor [42], which could also account for the reduced Cftr expression level induced by mifepristone. However, Cftr activity in Ussing chambers was not affected by mifepristone and Cftr activity was restored to control levels in the presence of dexamethasone.

Measurements in human bronchial submucosal gland-derived Calu-3 cells showed a reduction of CFTR mRNA expression by approximately 20% induced by 100 nM dexamethasone. In accordance to our results, Prota and colleagues, 2012 reported that dexamethasone reduced CFTR mRNA expression in Calu-3 cells by 30% [43]. The same study showed that dexamethasone increased CFTR protein expression two-fold which was attributed to an altered chaperone interaction with CFTR resulting in increased protein trafficking [43]. We were able to confirm the reduction of mRNA expression in Calu-3 cells and, in addition, demonstrated an increased CFTR activity in Ussing chamber measurements. CFTR activity was increased to 130% as indicated by the forskolin-induced I_SC_ and to approximately 180% as indicated by the glibenclamide-sensitive I_SC_. Furthermore, no increase of forskolin-induced I_SC_ by dexamethasone was detected when Cl^-^ was omitted in the Ussing chamber solution. By contrast, measurements in HCO_3_^-^ free solution resulted in a significantly increased forskolin-induced I_SC_ stimulated by dexamethasone. Therefore, dexamethasone seems to increase only the Cl^-^ transport in Calu-3 cells. Different aspects might explain the discrepancy between forskolin-induced HCO_3_^-^ and Cl^-^ transport. First, CFTR has a HCO_3_^-^ permeability of around 20% compared to Cl^-^ [44], so differences in CFTR HCO_3_^-^ conductance might not be easily detectable. Furthermore, Calu-3 cells apically express a Cl-/HCO_3_^-^ exchanger of the SLC26A family which is also stimulated by forskolin [45]. It is unknown if this anion exchanger is affected by GCs, but its activity might contribute to the measured current, as it also secretes HCO_3_^-^ into the apical lumen. In addition, some studies suggested that CFTR displays a “dynamic” selectivity and can switch between Cl^-^ permeable and HCO_3_^-^/Cl^-^ permeable states [46,47]. This is interesting since one study showed that glutamate exclusively elicits Cl^-^, but not HCO_3_^-^ conductance in the human sweat duct [46]. However, these results are controversial [48] and it is not known if GCs stimulate a “dynamic” selectivity. Finally, Cl^-^ entry into the cells might also affect Cl^-^ secretion and it is yet unknown if GCs alter apical or basolateral Cl^-^ or HCO_3_^-^ entry pathways.

In accordance to results obtained with Calu-3 cells, CFTR mRNA expression was also reduced by GCs in human bronchial epithelial 16HBE14o- cells, resulting in a 50% lower CFTR expression. Furthermore, treatment of primary rat airway epithelial cells with dexamethasone reduced Cftr mRNA expression by approximately 60% and increased CFTR activity to 150% for the forskolin-induced I_SC_ and 181% for the CFTR_172_inh-sensitive I_SC_. Therefore, the response to GCs seems to be conserved among airway-derived and alveolar cells.

Inhibition of the GR by mifepristone slightly reduced CFTR mRNA expression in Calu-3 cells but not to a significant extent. Furthermore, dexamethasone did not reduce CFTR expression in the presence of mifepristone. Therefore, GR inhibition itself has a small effect on CFTR expression but prevents the CFTR reduction induced by dexamethasone. Mifepristone also prevented the dexamethasone-induced increase of CFTR activity. Therefore, both the decrease of CFTR mRNA expression and the increase of CFTR activity induced by dexamethasone are mediated by the GR. In agreement with these results a study reported that the dexamethasone-induced occupancy of multiple GR elements causes the repression of CFTR expression in 16HBE14o- cells [49].

**Fig 11:**
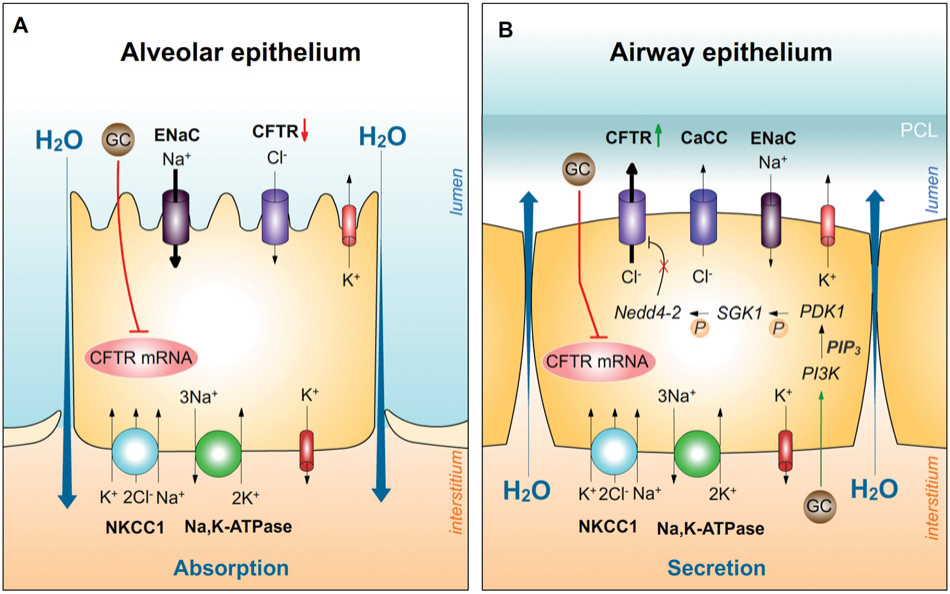
Effect of GCs on CFTR expression and activity in alveolar and airway epithelial cells. **A:** Postnatal alveolar cells predominantly exhibit Na^+^ absorption. Na^+^ enters the alveolar cells through apical Na^+^ channels like ENaC and is actively extruded basolaterally by the Na,K-ATPase. Thereby an osmotic gradient is generated that drives fluid absorption from the alveolar lumen into the interstitium. Apical CFTR is suggested to mainly absorb Cl^-^ in postnatal alveolar cells. GCs were shown to markedly reduce CFTR mRNA expression and activity in alveolar cells. **B:** In airway epithelial cells, Cl^-^ enters the basolateral membrane across NKCC1 and Na^+^ is actively extruded by the Na,K-ATPase. K^+^ channels recycle K^+^ at the basolateral side hyperpolarizing the membrane which represents the driving force for apical Cl^-^ extrusion through CFTR and Ca^2+^-dependent Cl^-^ channels (CaCC). Vectorial Cl^-^ transport drives water secretion into the airways. GCs reduced CFTR mRNA expression, but increased CFTR activity which supposedly depends on the proposed kinase pathway. GCs activate the PI3K leading to the generation of phosphatidylinositol (3,4,5)-trisphosphate (PIP_3_). Phosphoinositide-dependent kinase-1 (PDK1) is activated and further phosphorylates and activates SGK1. SGK1 in turn interacts with the ubiquitin-ligase Nedd4-2 reducing the affinity of Nedd4-2 for CFTR. Thereby the endocytic retrieval of CFTR is inhibited and CFTR plasma membrane abundance increased. PCL = periciliary liquid layer. Not all potentially important ion transporters and kinases are included in this schematic model.

In addition, LY-294002 also prevented the increase of CFTR activity induced by dexamethasone because no difference was observed between LY-294002-treated and LY-294002/dexamethasone-treated Calu-3 cells. LY-294002 also strongly reduced basal CFTR activity suggesting that the PI3K is a major regulator of CFTR activity. LY-294002 has been reported to inhibit the forskolin-induced phosphorylation of CFTR by protein kinase A and C and the forskolin-stimulated CFTR trafficking to the plasma membrane of duodenal epithelial cells [50]. This might explain the CFTR current reduction observed in the presence of LY-294002 in the basal as well as dexamethasone-stimulated I_SC_. In addition to the PI3K, the serum and glucocorticoid-dependent kinase 1 (SGK1) increases Na^+^ absorption by modulating the inhibition of ENaC by the ubiquitin ligase neural precursor cell expressed, developmentally down-regulated 4–2 (Nedd4-2) [51]. The same mechanism was proposed for CFTR as SGK1 enhances the functional activity of CFTR when coexpressed in *Xenopus* oocytes [52,53]. Studies further demonstrated that dexamethasone elevates the functional expression of wildtype (wt)- and ΔF508-CFTR and inhibition of either GR or PI3K and knock-down of SGK1 blocks the stimulating effect of dexamethasone on CFTR trafficking in pancreatic cells [54]. Moreover, in the transformed CF bronchial epithelial cell line CFBE41o- the GC-induced increase of SGK1 protein abundance enhanced ΔF508-CFTR [55] and wt-CFTR membrane abundance by inhibiting their endocytic retrieval [56]. CFTR was also shown to be involved in the regulation of stretch-induced proliferation in the lung by controlling airway smooth muscle contractions [57]. The effect of CFTR on muscle contraction was blocked by LY-294002 suggesting that CFTR-dependent proliferation proceeds by a pathway dependent on PI3K and protein kinase CK2 (also known as casein kinase 2) [57]. Therefore our results confirm a critical involvement of the PI3K in the regulation of CFTR activity. However the question remains why this effect on CFTR observed in airway-derived cells is not detected in alveolar cells. The reason for this might be the massive reduction of CFTR/Cftr mRNA expression in alveolar cells compared to the smaller response of bronchial CFTR expression that even a stimulating effect of GC possibly mediated by the PI3K in alveolar cells cannot measurably increase CFTR activity. Fig 11 summarizes the observed effects of GCs on CFTR/Cftr expression and activity in alveolar and airway-derived cells and proposes a potential pathway leading to an increased CFTR/Cftr activity in airway cells involving the kinases discussed above.

Finally we analyzed the impact of GCs on alternative Cl^-^ transporters in alveolar and bronchial cells. ANO1 dominates the Ca^2+^-dependent Cl^-^ currents in pulmonary epithelia and is expressed at the apical membrane next to CFTR [58]. ANO1/Ano1 mRNA expression was strongly reduced by dexamethasone in alveolar cells whereas ANO1 expression was increased in bronchial 16HBE14o- and Calu-3 cells. The reason for this differential regulation of ANO1/Ano1 by GCs is yet unknown. We suggest that the relevance of this effect might be due to the physiological function of the analyzed cells. During and after birth the main task of alveolar cells is the absorption of fluid to promote air breathing while cells of the bronchial glands play a major role in secretion of the airway lining fluid. If ANO1/Ano1 participates in Cl^-^ secretion the reduction in alveolar cells and the increase in bronchial cells induced by GCs possibly promotes the physiological task of both cell types. It is unknown if ANO1 is developmentally regulated in the same manner as CFTR, yet the demonstrated hormonal regulation of ANO1/Ano1 suggests a physiological function during development. In addition, Ano1 knock-out mice display a pronounced tracheomalacia, accumulate mucous in the tracheal lumen and die during the early postnatal period [59,60] further delineating an important function of ANO1 during fetal development. CLC5 and ANO6 are described as components of the outwardly rectifying Cl^-^ channels (ORCC) [58,61,62]. Both ANO6/Ano6 and CLC5/Clc5 mRNA expression were up-regulated by dexamethasone in alveolar cells, whereas in bronchial 16HBE14o- and Calu-3 cells the expression of the ORCC components was not affected. The anoctamine family consists of at least 10 members and it is known that in addition to ANO1 and ANO6, ANO2 and ANO7 show Cl^-^ channel activity (see review [63]). A hormonal regulation of ANO2 and ANO7 has yet to be determined. Finally, NKCC1/Nkcc1 mRNA expression was strongly reduced by dexamethasone in alveolar and also in bronchial cells. Since it has been shown that prenatal fluid and Cl^-^ secretion is dependent on NKCC1 function, the results demonstrate a reduction of secretion possibly to enable perinatal fluid absorption.

Taken together the results show a differential regulation of CFTR/Cftr by GCs (Fig 11). CFTR/Cftr mRNA expression was reduced in alveolar and bronchial cells yet not to the same extent and CFTR/Cftr expression in alveolar cells is more responsive to GC treatment. The reason for this is yet unknown but postnatal alveolar cells are mainly Na^+^ absorptive whereas airway cells still secrete Cl^-^. The cause for the differential effect of GC on CFTR/Cftr channel activity has yet to be determined but transcriptional and post-translational effects seem to play a part. In summary, the study provides three conclusions. The first conclusion proposes a physiological correlation between the GC concentration and CFTR expression and function in the fetus and thereby possibly marks the switch from lung proliferation to epithelial differentiation. The second conclusion suggests that GCs not only increase absorption during perinatal lung transition but simultaneously diminish secretion by reducing alveolar CFTR. The third conclusion proposes that the CFTR reduction induced by GCs further enables ENaC activity and thereby enhances perinatal fluid absorption.
